# HEARTBREAK Controls Post-translational Modification of INDEHISCENT to Regulate Fruit Morphology in *Capsella*

**DOI:** 10.1016/j.cub.2020.07.055

**Published:** 2020-10-05

**Authors:** Yang Dong, Mateusz Majda, Jan Šimura, Robert Horvath, Anjil K. Srivastava, Łukasz Łangowski, Tilly Eldridge, Nicola Stacey, Tanja Slotte, Ari Sadanandom, Karin Ljung, Richard S. Smith, Lars Østergaard

**Affiliations:** 1 Crop Genetics Department, John Innes Centre, Norwich NR4 7UH, UK; 2 Cell and Developmental Biology Department, John Innes Centre, Norwich NR4 7UH, UK; 3 Umeå Plant Science Centre, Department of Forest Genetics and Plant Physiology, Swedish University of Agricultural Sciences, 901 83 Umeå, Sweden; 4 Department of Ecology, Environment and Plant Sciences, Science for Life Laboratory, Stockholm University, 106 91 Stockholm, Sweden; 5 Department of Biosciences, University of Durham, Durham DH1 3LE, UK

**Keywords:** fruit morphology, *Capsella rubella*, post-translational modification, SUMOylation, gene expression, anisotropic cell growth

## Abstract

Morphological variation is the basis of natural diversity and adaptation. For example, angiosperms (flowering plants) evolved during the Cretaceous period more than 100 mya and quickly colonized terrestrial habitats [1]. A major reason for their astonishing success was the formation of fruits, which exist in a myriad of different shapes and sizes [2]. Evolution of organ shape is fueled by variation in expression patterns of regulatory genes causing changes in anisotropic cell expansion and division patterns [3, 4, 5]. However, the molecular mechanisms that alter the polarity of growth to generate novel shapes are largely unknown. The heart-shaped fruits produced by members of the *Capsella* genus comprise an anatomical novelty, making it particularly well suited for studies on morphological diversification [6, 7, 8]. Here, we show that post-translational modification of regulatory proteins provides a critical step in organ-shape formation. Our data reveal that the SUMO protease, HEARTBREAK (HTB), from *Capsella rubella* controls the activity of the key regulator of fruit development, INDEHISCENT (CrIND in *C. rubella*), via de-SUMOylation. This post-translational modification initiates a transduction pathway required to ensure precisely localized auxin biosynthesis, thereby facilitating anisotropic cell expansion to ultimately form the heart-shaped *Capsella* fruit. Therefore, although variation in the expression of key regulatory genes is known to be a primary driver in morphological evolution, our work demonstrates how other processes—such as post-translational modification of one such regulator—affects organ morphology.

## Highlights

• *HTB* encodes a SUMO protease required for fruit shape in *Capsella*
• Anisotropic cell growth is suppressed in the fruit valves of the *htb* mutant
• HTB stabilizes CrIND through de-SUMOylation to facilitate local auxin biosynthesis

## Graphical Abstract

**Figure:**
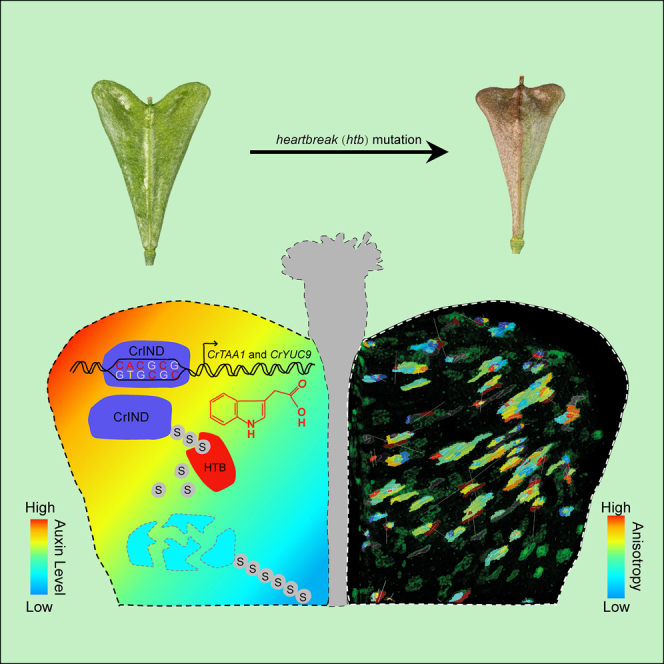

## Teaser

Dong et al. reveal post-translational modification as a so-far-undisclosed driver of morphological diversity. They show that the SUMO protease HEARTBREAK is required to de-SUMOylate a key regulator of fruit-shape determination in *Capsella*, thereby initiating a transduction pathway leading to local auxin biosynthesis and anisotropic cell growth.

## Results and Discussion

### The *heartbreak* (*htb*) Mutant Has Valve Growth Defects and Reduced Cell-Growth Anisotropy

Organs in multicellular organisms have evolved into specific shapes that are critical for their function. Accordingly, little diversity is observed in organ morphology between individuals of the same species, with organs consistently and robustly developing into specific shapes [9]. By contrast, major variation in organ shape can exist between closely related species, as observed for fruits, leaves, insect wings, or the outer ears of mammals [7, 10, 11, 12]. Changes in the expression pattern of key regulatory genes is a major driver of such morphological diversity, ultimately giving rise to changes in cell division patterns and cell expansion [13, 14]. We have shown that sequence variation in regulatory domains of the fruit-tissue identity gene, *INDEHISCENT* (*IND*) (*CrIND* in *Capsella*), is responsible for augmentation of its expression domain in the heart-shaped fruits from *Capsella rubella*. In turn, CrIND induces expression of auxin biosynthesis genes required for growth of the shoulders of the heart [8].

**Figure 1:**
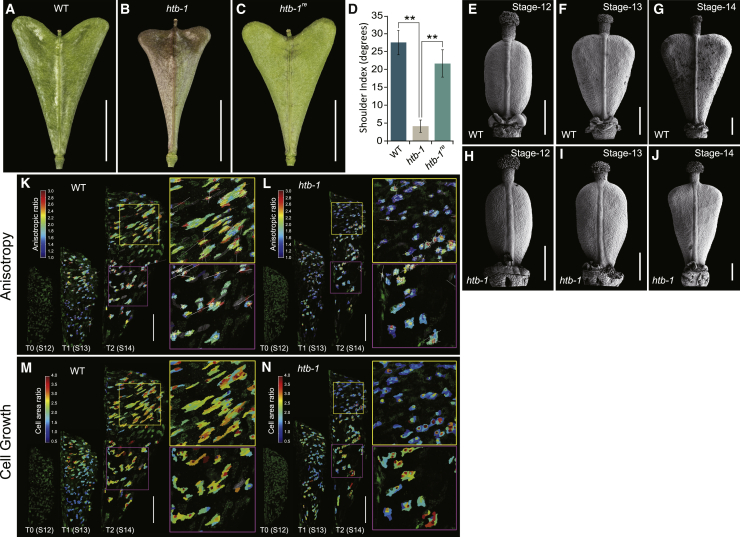
The *htb* Mutant Produces Fruits with Defective Fruit Shape and Reduced Anisotropic Cell Growth (A*–*C) Fruit morphology of WT (A), *htb-1* (B), and rescue line of *htb-1* transformed with *pHTB:HTB:GFP* (C) at stage 17. (D) Shoulder index measurements of fruits from WT, *htb-1*, and *htb-1*^*re*^ (*pHTB:HTB:GFP htb-1*) plants. (E–G) Scanning electron microscopy (SEM) images of fruits from WT at developmental stages 12 (E), 13 (F), and 14 (G), showing fruit-shoulder growth after pollination. (H–J) SEM images of fruits from *htb-1* at stages 12 (H), 13 (I), and 14 (J), showing compromised development of the fruit shoulders. (K–N) Time-lapse live imaging of developing fruits from stages 12 to 13 and 13 to 14 in WT (K and M) and *htb-1* mutant (L and N). Cells are outlined by RFP signal of the clonal sectors derived from heat-shock treatment of *pHS:CRE/BOB-lox* line. The heatmaps represent the anisotropy (K and L) and the overall cell area ratio (M and N). Scale bars, (A–C) 5 mm; (E–N) 100 μm. Error bars in (D) represent SD of 30 individual fruits. ^∗∗^p < 0.01 (Student’s t test). See also Figure S1.

To identify genetic factors controlling this process and required for the formation of heart-shaped fruits in *Capsella*, we carried out a forward genetic screen of an ethyl methanesulfonate (EMS)-induced *Capsella rubella* (Cr22.5) mutant population. One mutant, *heartbreak* (*htb*), was isolated because of its strong defects in fruit development with compromised outgrowth of the fruit shoulders (Figures 1A, 1B, and 1D). Moreover, compared with wild type (WT), the *htb* mutant exhibits defects throughout both vegetative and reproductive development (Figures S1A–S1J). This demonstrates that the *HTB* gene regulates multiple developmental processes.

In WT *Capsella*, the heart-shaped fruit develops from a disc-formed (ovate spheroid) gynoecium soon after pollination [6] (Figure 1E). From stage 13 onward, directional outgrowths of the apical parts of the valves found formation of the heart shape by stage 14 (Figures 1F and 1G; developmental stages defined in [8]). Comparative ontogenetic analysis revealed no defects between WT and *htb* during early gynoecium development (Figures 1E and 1H). In contrast to WT, however, the outgrowth of the *htb* valve apex is significantly suppressed from stage 13 (Figures 1F, 1G, 1I, and 1J).

During postfertilization development, anisotropic cell expansion drives fruit growth toward the final size and shape [7, 15]. To assess the cellular basis underlying the *htb* phenotype, we traced the cell growth dynamics by time-lapse imaging of developing fruits [16]. We chose three specific stages: stage 12 (immediately preceding the initiation of shoulder outgrowth); stage 13 (outgrowth begins); and stage 14 (shoulders are clearly formed; Figures 1E–1G). In stage-13 WT fruits, cells in the apical part of the valve grew anisotropically along the medio-lateral axis (Figures 1K and S1K). At stage 14, most of the cells in the apical part of the WT fruit had become highly anisotropic, growing toward the developing fruit shoulders, although cells in the basal part of the fruit remained largely isotropic from stages 12 to 14 (Figure 1K). In WT, the overall cell expansion rate was similar between apical and basal parts of the fruit (Figures 1M and S1L). In contrast to WT, cells in the valves of the *htb* mutant grew isotropically throughout all the stages studied here, leading to reduced growth rate in the shoulders (Figures 1L and S1K). Also, in comparison to WT, the *htb* mutant displayed a decreased overall cell expansion rate in the apical part of the fruit (Figures 1N and S1L). These data demonstrate that the *HTB* locus functions to promote anisotropic cell growth in the fruit valves.

### The *HTB* Gene Encodes a Nuclear-Localized Protein Annotated as a SUMO Protease

**Figure 2:**
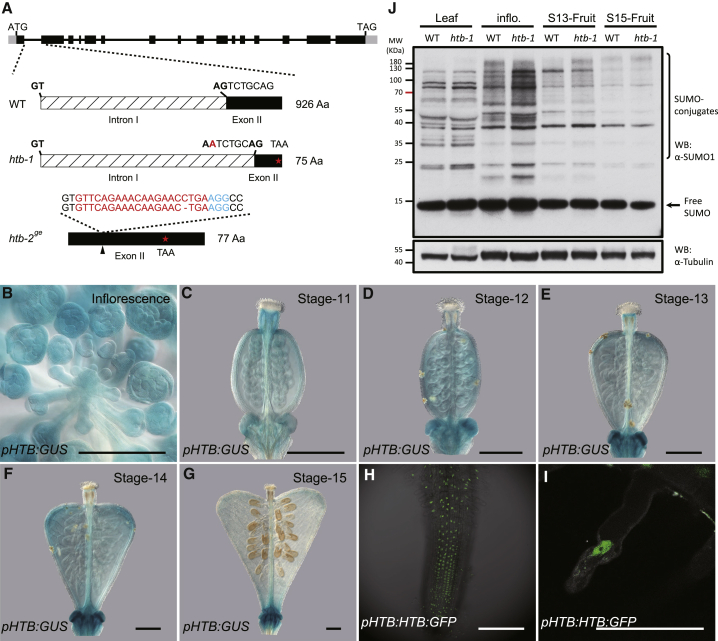
Molecular Cloning and Expression Analysis of *HTB* (A) Cloning of the *htb-1* allele identified a G-to-A mutation in the acceptor site of the first intron of Carubv10008238, which disrupts the splicing of the first intron and results in a 7-bp deletion in the second exon, generating a premature stop codon in exon 2. The *htb-2*^*ge*^ allele was generated by CRISPR with a single-base-pair deletion in the exon 2, resulting in a frameshift giving rise to a 77-amino-acid (aa) protein. The guide RNAs and PAM sequences were indicated by red and blue characters, respectively. (B–G) GUS staining of *pHTB:GUS* line showing the dynamic expression of *HTB* during fruit development. Uniform expression of *HTB* is detected in inflorescence tissue (B) and in the gynoecium at stage 11 (C) and 12 (D). A stronger *HTB* expression is detected in the developing fruit shoulders in stages 13 (E) and 14 (F). At stage 15, only residual *HTB* expression is observed in the fruit (G). (H and I) Subcellular localization of HTB:GFP protein in the roots of *pHTB:HTB:GFP* line. Scale bars in (B)–(I) represent 100 μm. (J) Comparative analysis of SUMO conjugates in total protein extracts from leaf, inflorescence (inflo.), and stage-13 (S13) and stage-15 (S15) fruits between WT and *htb-1*. The α-tubulin was immunoblotted as a loading control. See also Figure S2.

The *htb* mutation segregates as a single-locus recessive trait (Figure S2B). By whole-genome sequencing and associative mapping, we identified two candidate mutations in the predicted genes, *Carubv10012951* and *Carubv10008238*. A synonymous mutation in the first exon of *Carubv10012951* precluded it for further consideration. Instead, a potential causal mutation in the acceptor site of the first intron of a predicted gene, Carubv10008238, was investigated further. This gene encodes a putative small ubiquitin modifer (SUMO) protease, a member of the ULP2 subfamily of cysteine proteases, and is orthologous to the *Arabidopsis SPF1/ASP1* gene (Figure S2A) [17, 18]. The mutation disrupts the splicing of the first intron, which instead occurs after an alternative site 7 bp into the second exon, resulting in a frameshift and premature stop codon (Figures 2A and S2C). We will refer to this mutant allele as *htb-1*. Verification of the causality of this mutation on fruit shape was confirmed as follows: (1) expression of Carubv10008238 driven by its native promoter fully complemented the *htb-1* mutant (Figures 1C and 1D); (2) a knockout line of Carubv10008238 using CRISPR-Cas9, leading to a single-base deletion in the second exon (*htb-2*^*ge*^), phenocopied the *htb-1* fruit character alongside other developmental defects (Figures 2A and S2D–S2F); and (3) F_1_ plants of *htb-1* crossed with the *htb-2*^*ge*^ mutant show the same phenotype as *htb-1* (Figure S2G). Together, these experiments show that the developmental defects observed in the *htb-1* mutant are caused by loss of the Carubv10008238 gene, which we henceforth refer to as *HEARTBREAK* (*HTB*), encoding a putative SUMO protease.

In agreement with the wide range of developmental defects of the *htb-1* mutant, we found a *pHTB:GUS* reporter line to be expressed throughout plant development, including vascular tissue of cotyledons and roots and in root tips of seedlings (Figures S2H and S2I). *pHTB:GUS* signal seemed uniformly distributed in the inflorescences and young gynoecia (Figures 2B–2D). Notably, in the developing fruit, stronger *HTB* promoter activity is detected in the shoulders from stage 13 to stage 14, when the heart shape starts to develop, although at stage 15, only residual *HTB* expression is detected (Figures 2E–2G). *HTB* expression therefore correlates spatially and temporally with fruit growth in agreement with its role in promoting anisotropic cell growth in the valves.

The SPF1/ASP1 protein is located in the nucleus of *Arabidopsis* cells [18, 19]. To test the subcellular localization of HTB, we used a *pHTB:HTB:GFP* reporter line, which fully complements the *htb-1* mutant (Figures 1C and 1D). Strong GFP signal was seen specifically within the nucleus but excluded from the nucleolus in root cells (Figures 2H and 2I). A similar nuclear localization pattern was observed using transient overexpression of an HTB-GFP fusion protein in WT leaf protoplasts (Figure S2J). These data suggest that HTB exerts its function on fruit-shape formation by affecting the activity of nuclear proteins.

### SUMO Conjugate Levels Are Elevated in the *htb-1* Mutant

The SUMOylation of proteins is a dynamic process with reversibility in conjugation and deconjugation [20]. SUMO proteases falling into the class of ubiquitin-like proteases (ULPs) belong to the cysteine protease family and are able to mediate SUMO maturation as well as SUMO deconjugation from protein targets through their endopeptidase and isopeptidase activity, respectively [21]. In order to determine whether HTB affects SUMO-conjugation levels, we compared the SUMOylation profiles between WT and *htb-1* by western blotting using specific anti-SUMO1 antibodies. Compared with WT, high-molecular-weight SUMO conjugates constitutively accumulated in total-protein extracts from the *htb-1* mutant. This was particularly evident in inflorescence tissue and stage-13 fruits (Figure 2J), suggesting that the developmental defects observed in the *htb-1* mutant (Figures S1A–S1J) is due to over-SUMOylation of proteins that are targets of the HTB SUMO protease.

### HTB Controls Fruit Development by Regulating Auxin Biosynthesis

SUMO proteases have been reported to control SUMOylation levels of transcription factors, chromatin remodeling factors, and/or transcriptional co-repressors [18, 22, 23, 24]. In order to understand the relationship between the transcriptional profile and HTB function in fruit development, we performed a comparative transcriptomic analysis of stage-13 fruits between WT and *htb-1*, when the developmental difference started to emerge (Figures 1F and 1I). The RNA-profiling analysis generated a total of 605 significant differentially expressed genes (DEGs) between WT and *htb-1*. Among them, 190 were upregulated and 415 were downregulated (Data S1A and S1B). Gene Ontology (GO) and pathway-enrichment analyses identified enrichment of DEGs in processes such as oxidation-reduction, protein phosphorylation, responses to light stimulus, and cell wall organization and modification (Figures S3A and S3C; Data S2A and S2B). Intriguingly, genes involved in hormone response were over-represented in the DEGs, especially among the downregulated genes (Figures S3B and S3D; Data S2A and S2B). Among the 26 downregulated DEGs associated with hormone response, 11 were associated with auxin response, pinpointing a possible role of HTB in regulating auxin dynamics during fruit-shape determination (Figures S3D and S3E; Data S2B).

**Figure 3:**
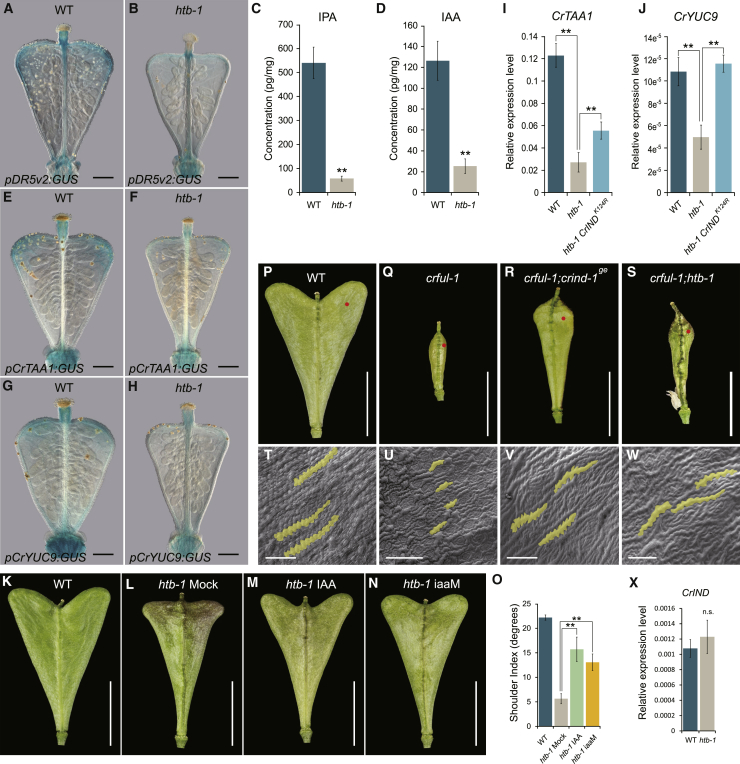
HTB Regulates Fruit Growth via Fine-Tuning Auxin Homeostasis (A and B) Auxin signaling visualized by *pDR5v2:GUS* in stage-14 fruits of WT (A) and *htb-1* (B). (C and D) Measurements of IPA (C) and IAA (D) in fruit shoulders of WT and *htb-1* in stage-14 fruits. (E–H) Expression of *CrTAA1* and *CrYUC9* shown by GUS staining of the *pCrTAA1:GUS* and *pCrYUC9:GUS* reporter lines at developmental stage 14 in WT (E and G) and *htb-1* (F and H). (I and J) Expression analysis of *CrTAA1* (I) and *CrYUC9* (J) in fruit shoulders of WT, *htb-1*, and *htb-1 pCrIND:CrIND*^*K124R*^*:GFP* at stage 14. (K–N) Fruit morphology of WT (K), IAA mock (L), or IAA (M) treatment on *htb-1* and *htb-1 pCrIND:iaaM* (N) at stage 17. (O) Shoulder index measurements of fruits from WT, *htb-1* ± IAA treatment, and *htb-1 pCrIND:iaaM* plants. (P–S) Fruit morphology of WT (P), *crful-1* (Q), *crful-1;crind-1*^*ge*^ (R), and *crful-1;htb-1* (S) at stage 17. Red dots indicate the location from where SEMs were taken in (T)–(W). (T–W) SEM images of valve epidermal cells of WT (T), *crful-1* (U), *crful-1;crind-1*^*ge*^ (V), and *crful-1;htb-1* (W) at stage 17. (X) Expression analysis of *CrIND* in stage-14 fruits of WT and *htb-1*. n.s. indicates no statistically significant difference from WT. Scale bars in (A), (B), and (E)–(H), 150 μm; (K)–(N) and (P)–(S), 5 mm; and (T)–(W), 50 μM. Error bars in (C), (D), (I), (J), and (X) represent SD of three biological replicates and in (O) represent SD of 30 individual fruits. ^∗∗^p < 0.01 (Student’s t test). See also Figure S3 and Data S1 and S2.

We recently reported that the development of the heart-shaped *Capsella* fruit requires an auxin maximum in the fruit shoulders ensured by local expression of auxin biosynthesis genes, *CrTAA1* and *CrYUC9* [8]. Hence, we analyzed whether auxin dynamics was disrupted in the *htb-1* fruits compared to WT. To visualize the auxin signaling pattern in the fruit valves, we used the *pDR5v2:GUS* reporter whose expression marks and precedes shoulder growth and introduced it into *htb-1*. In stage-14 WT fruit, a gradient of auxin signaling was observed in the valves with a maximum in the fruit shoulders (Figure 3A). In contrast, in the *htb-1* mutant, the auxin maxima in the shoulders were lost, signifying a reduction of auxin response in the *htb-1* fruits (Figure 3B). We next asked whether the lack of auxin maxima in the *htb-1* fruit shoulders was due to low auxin levels. Direct measurements of both the predominant natural auxin, indole-3-acetic acid (IAA), and its precursor, indole-3-pyruvate (IPA), showed a significant reduction in the shoulders of *htb-1* compared to WT (Figures 3C and 3D). In correlation with reduced IPA and IAA levels, we found that expression of *CrTAA1* and *CrYUC9* was lower in the *htb-1* fruit shoulders compared to WT (Figures 3E–3J). These data suggest that the decrease in auxin response observed in *htb-1* fruits can be attributed to low levels of auxin biosynthesis, resulting from reduced *CrTAA1* and *CrYUC9* expression. Rescue of the *htb-1* phenotype by exogenous application of IAA and valve-shoulder-specific expression of a bacterial auxin biosynthesis gene (*pCrIND:iaaM*) provided further evidence that auxin biosynthesis is a downstream output of HTB activity required for fruit-shape formation (Figures 3K and 3O).

### HTB Controls CrIND Function by De-SUMOylation

**Figure 4:**
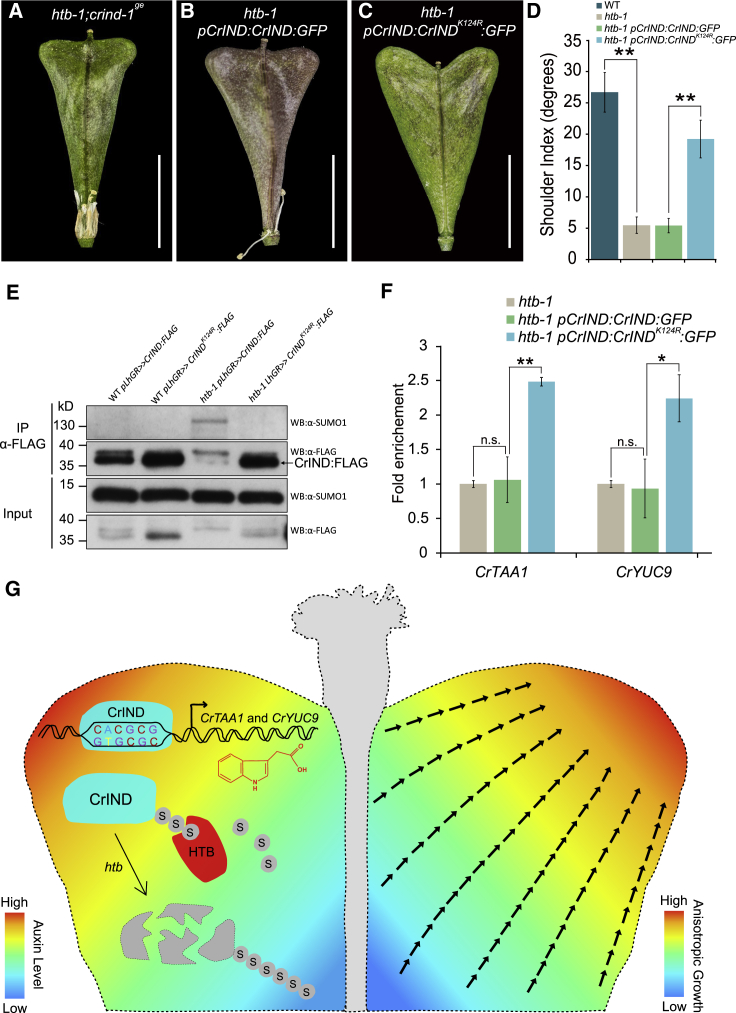
HTB Stabilizes CrIND by De-SUMOylation (A–C) Fruit morphology of *htb-1;crind-1*^*ge*^ (A), *htb-1 pCrIND:CrIND:GFP* (B), and *htb-1 pCrIND:CrIND*^*K124R*^*:GFP* (C) at stage 17. (D) Shoulder index measurements of fruits from WT, *htb-1*, *htb-1 pCrIND:CrIND:GFP*, and *htb-1 pCrIND:CrIND*^*K124R*^*:GFP* plants. (E) SUMOylation status of CrIND protein using *pLhGR≫CrIND:FLAG* and *pLhGR≫CrIND*^*K124R*^*:FLAG* lines. Immunoprecipitation experiments were conducted using anti-FLAG beads. Immunoblots were probed with anti-FLAG or anti-SUMO1 antibodies. (F) Chromatin immunoprecipitation (ChIP) analysis of CrIND/CrIND^K124R^ associated with the *CrYUC9* and *CrTAA1* promoters. (G) Model for the control of heart-shape fruit development by CrIND and HTB in *Capsella*. Precise production of auxin in the tips of fruit shoulders controlled by CrIND induces anisotropic cell growth in the valves in a direction toward the shoulder tips. CrIND protein is de-SUMOylated by HTB, whereas in the *htb-1* mutant, CrIND is SUMOylated and destabilized, thereby reducing its ability to activate expression of auxin biosynthesis gene (*CrTAA1* and *CrYUC9*). Scale bars, (A–C) 5 mm. Error bars in (D) represent SD of 30 individual fruits and in (F) represent SD of three biological replicates. n.s. indicates no statistically significant difference from *htb-1*, *p < 0.05 and ^∗∗^p < 0.01 (Student’s t test). See also Figure S4.

In *Capsella*, shoulder-specific expression of *CrTAA1* and *CrYUC9* is regulated by the basic-helix-loop-helix (bHLH) transcription factor, CrIND [8]. In the *crind-1*^*ge*^ mutant, the fruit shoulders fail to fully expand due to depletion of auxin in the fruits compared to WT [8]. The *htb-1* mutant exhibits a similar phenotype as *crind-1*^*ge*^, and lack of an obvious exacerbation of the single mutant phenotypes in the *htb-1 crind-1*^*ge*^ double mutant suggests that *HTB* and *CrIND* function in the same pathway (Figure 4A). To explore this possibility further, we crossed *htb-1* with the *crful-1* mutant previously shown to be partially rescued by mutations in the *CrIND* gene [8] (Figures 3P–3R, 3T–3V, and S3F). The *htb-1* mutant also partially rescues the strong growth defect of *crful-1*, although to a lesser extent than *crind-1*^*ge*^ (Figures 3S, 3W, and S3F). It is therefore possible that the absence of HTB partially overturns the effect of ectopic *CrIND* expression previously reported to occur in *crful-1* [8].

Interestingly, expression of *CrIND* was unchanged in *htb-1* compared to WT (Figure 3X). This led us to test whether CrIND function is regulated post-translationally by HTB through SUMOylation. In plant cells, SUMOylation occurs through an isopeptide-bond formation between the di-glycine at the C-terminal of the SUMO peptide and the accessible lysyl ε-amino group within the targets [25]. Cumulative SUMO target datasets suggest a consensus ψ-K-X-D/E canonical SUMOylation motif (ψ, hydrophobic amino acid; X, any amino acid) [26]. Searching the CrIND sequence identified a consensus SUMO motif in amino acid positions 123–126 (AKMD) with lysine in position 124 (K124) as a potential SUMO-conjugation residue. To investigate the functional relevance of K124 in CrIND with HTB, we produced a mutant variant of CrIND, in which K124 is mutated to the related but unSUMOylatable amino acid, arginine (R), and compared the function of CrIND and CrIND^K124R^ in *htb-1* background. The K124R mutation did not change the protein function, as both *pCrIND:CrIND:GFP* and *pCrIND:CrIND*^*K124R*^*:GFP* fully rescued the *crind* mutant (Figures S4A–S4D). In the *htb-1* background, however, we observed a different behavior of these two proteins. Although *pCrIND:CrIND:GFP* failed to complement the *htb-1* mutant, *pCrIND:CrIND*^*K124R*^*:GFP* effectively rescued the fruit defects of *htb-1*, developing fully heart-shaped fruits (Figures 4B–4D). This implies that post-translational modification of the K124 residue in CrIND is the primary cause of the defect in *htb-1* fruit-shoulder growth and suggests that HTB functions to de-SUMOylate CrIND on this residue.

We then tested whether CrIND SUMOylation status depends on HTB. To this end, overexpression of FLAG-tagged versions of *CrIND* and *CrIND*^*K124R*^ in WT and the *htb-1* mutant was achieved using a two-component dexamethasone (DEX)-inducible system (Figure S4E). A pull-down experiment of FLAG-tagged CrIND/CrIND^K124R^ detected a high-molecular-weight version of SUMOylated CrIND only in the *htb-1* mutant background (Figure 4E). Moreover, western blotting with FLAG antibody revealed low abundance of CrIND in *htb-1* compared to WT, whereas no reduction was observed with the CrIND^K124R^ version in *htb-1* (Figure 4E). These data demonstrate that HTB positively controls CrIND levels through de-SUMOylation, suggesting that SUMOylation on K124 of CrIND leads to its destabilization.

In agreement with reduced stability of CrIND in the *htb-1* mutant, chromatin immunoprecipitation (ChIP) assays in *htb-1* revealed that promoter regions of *CrTAA1* and *CrYUC9* were less enriched with CrIND-GFP compared to CrIND^K124R^-GFP (Figure 4F). On the other hand, the binding affinities to *CrTAA1* and *CrYUC9* promoters were not significantly different between CrIND-GFP and CrIND^K124R^-GFP when ChIP assays were carried out in the *crind-1*^*ge*^ background (Figure S4F). Furthermore, *CrTAA1* and *CrYUC9* expression in the fruit shoulders is restored in *htb-1* carrying the *pCrIND:CrIND*^*K124R*^*:GFP* transgene (Figures 3I and 3J). Together, these biochemical and genetic data demonstrate that HTB acts directly on CrIND, leading to local expression of auxin biosynthesis genes. Although the effect of SUMOylation can vary widely between proteins, our results align with observations in both plants and animals that SUMOylation of transcription factors affects their stability and activity toward target genes [22, 23, 24, 27].

Our analyses did not identify any morphological differences between fruits from *crind* plants expressing *pCrIND:CrIND:GFP* and *pCrIND:CrIND*^*K124R*^*:GFP* (Figures S4C and S4D). Although we cannot rule out subtle defects in other processes under CrIND control, this suggests that SUMOylation-resistant CrIND functions identically to wild-type CrIND, thus raising the question as to the purpose of the SUMOylation motif. We have previously shown that ectopic expression of *IND* in *Arabidopsis* can lead to profound developmental defects [28, 29]. As the expression domain of *CrIND* expanded to the tip of fruit valves in *Capsella*, we speculate that SUMOylation provides an additional regulatory layer to control CrIND activity to prevent deleterious effects.

### HTB Protein Function Is Conserved between *Capsella* and *Arabidopsis*

In *Arabidopsis*, mutations in *SPF1/ASP1*, the ortholog of *HTB*, result in delayed flowering and abnormal floral and ovule development, although no fruit defect was described [17, 18, 19]. We therefore asked whether the *HTB* function in relation to fruit development is unique to *Capsella*. To this end, we transformed *htb-1* with constructs of SPF1/ASP1 genomic sequences driven by the native *HTB* promoter (*pHTB*:*AtSPF1/ASP1*). The *pHTB*:*AtSPF1/ASP1* construct fully complemented the *htb-1* fruit defects in a similar manner to the *pHTB*:*HTB* construct, showing that HTB and SPF1/ASP proteins are functionally conserved (Figures S4G–S4J). This scenario is further supported by a population genetics analysis on the *HTB* locus in *C. grandiflora*, which is an out-crossing member of the *Capsella* genus [30]. In *C. grandiflora*, the protein sequence of *HTB* has been subjected to purifying selection (ω [dN/dS] < 1) [31], signifying no evidence for neo-functionalization of the HTB proteins (Figure S4K). Therefore, the difference in fruit shape between *Capsella* and *Arabidopsis* is not caused by functional diversification of the HTB protein itself. Rather, HTB is more likely to have been recruited specifically in the *Capsella* genus to modulate CrIND protein function, leading to precise auxin production and specific anisotropic cell expansion to form the heart-shaped *Capsella* fruit (Figure 4G).

### Concluding Remarks

In this study, we showed that the SUMO protease HTB targets the bHLH transcription factor CrIND for de-SUMOylation on lysine residue, K124. Removal of CrIND SUMOylation by HTB is required to stabilize CrIND and allow local activation of auxin biosynthesis genes in the fruit valves (Figure 4G). This, in turn, leads to stimulation of anisotropic cell expansion and formation of the heart-shaped *Capsella* fruit (Figure 4G). Although variation in the expression of key regulatory genes is known to be a primary driver in controlling morphological evolution, we demonstrated here how a post-translational modification of one such regulator, CrIND, affects organ morphology.

## STAR★Methods

### Key Resources Table

**Table:** 

REAGENT or RESOURCE	SOURCE	IDENTIFIER
**Antibodies**		

Anti-GFP monoclonal antibody	Roche	RRID: AB_390913
Mouse monoclonal [M2] anti-FLAG-HRP antibody	Abcam	RRID: AB_869428
Rabbit polyclonal anti-SUMO1 antibody	Abcam	RRID: AB_2198088
Rabbit anti-mouse IgG-HRP secondary antibody	Abcam	RRID: AB_955440
Goat anti-rabbit IgG-HRP secondary antibody	Abcam	RRID: AB_955447
Mouse monoclonal Anti-α-tubulin antibody	Sigma	RRID: AB_477579

**Bacterial Strains**		

DH5-alpha competent *E. coli*	NEB	C29871
*Agrobacterium tumefaciens* strain LBA4404	N/A	N/A

**Biological Samples**		

*Capsella rubella* (WT, 22.5)	[6]	N/A
*pHS:CRE/BOB-lox*	[6]	N/A
*pDR5v2*:GUS	[8]	N/A
*pCrTAA1*:GUS	[8]	N/A
*pCrYUC9*:GUS	[8]	N/A
*pCrIND:iaaM*	[8]	N/A
*crind-1*^*ge*^*pCrIND:CrIND:GFP*	[8]	N/A
*htb-1*	This paper	N/A
*htb-2*^*ge*^	This paper	N/A
*crind-1*^*ge*^	[8]	N/A
*crful-1*	[6]	N/A
*crful-1; crind-1*^*ge*^	[8]	N/A
*htb-1; crind-1*^*ge*^	This paper	N/A
*htb-1; crful-1*	This paper	N/A

**Chemicals Peptides, and Recombinant Proteins**		

Phusion High-Fidelity DNA polymerase	NEB	M0530L
DnaseI	QIAGEN	79254
In-Fusion Cloning Recombinase	Clontech	638909
Protease Inhibitor Cocktail	Roche	11836170001
PMSF	Roche	10837091001
Dexamethasone	Sigma-Aldrich	D4902
Indole-3-acetic acid (IAA)	Sigma-Aldrich	I5148
Ethyl methanesulphonate	Sigma-Aldrich	M0880
N-Ethylmaleimide	Sigma-Aldrich	04259
DMSO	Sigma-Aldrich	D8418
Gibberellin	Sigma-Aldrich	G7645
Hygromycin	Roche	10843555001
DL-phosphinothricin	Duchefa	P0519.0250
Formaldehyde	Sigma-Aldrich	F8775
Cellulase R10	Yakult	190517
Macerozyme R10	Yakult	131126
Tween-20	Sigma-Aldrich	P9406
Triton X-100	Sigma-Aldrich	T8787
X-gluc	MELFORD	MB1121
Oligonucleotides	List given in Table S1	N/A

**Critical Commercial Assays**		

QIAprep Spin MiniPrep Kit	QIAGEN	27104
DNeasy Plant Mini Kit	QIAGEN	69104
QIAquick PCR Purification Kit	QIAGEN	28104
RNeasy Plant Mini Kit	QIAGEN	74904
Pierce Protein G Magnetic Beads	ThermoFisher	19958500
Pierce ECL Western Blotting Substrate	ThermoFisher	32209
Anti-FLAG [M2] Magnetic Beads	Sigma-Aldrich	M8823
SuperScript IV First-Strand Synthesis System	ThermoFisher	18091050
SYBR Green JumpStart Taq ReadyMix	Sigma-Aldrich	S4438

**Recombinant DNA**		

*pHTB*:*GUS*	This Paper	N/A
*pHTB*:*HTB*	This Paper	N/A
*pHTB:HTB:GFP*	This Paper	N/A
*pHTB:AtSPF1/ASP1*	This Paper	N/A
*pCrIND:CrIND*^*K124R*^*:GFP*	This Paper	N/A
*pLhGR≫CrIND:FLAG*	This Paper	N/A
*pLhGR≫CrIND*^*K124R*^*:FLAG*	This Paper	N/A

**Software and Algorithms**		

MorphoGraphX	[16]	https://www.mpipz.mpg.de/MorphoGraphX
CRISPR-2.0	[32]	http://crispr.hzau.edu.cn/CRISPR2/
CLUSTAL-X	[33]	http://www.clustal.org/clustal2/
MEGA5	[34]	https://www.megasoftware.net/

**Other**		

PVDF membrane	GE Healthcare	10600021
Miracloth	Merck	475855
X-ray film	Kodak	4741019289

### Resource Availability

#### Lead Contact

Further information and requests for resources and reagents should be directed to and will be fulfilled by the Lead Contact, Lars Østergaard (lars.ostergaard@jic.ac.uk).

#### Materials Availability

• Plasmids and germplasm generated in this study is available upon request.

#### Data and Code Availability

• Data from genome sequencing and RNA-Seq have been deposited with the European Bioinformatics Institute (EBI): BioProject ID: PRJEB39302
Title: ena-STUDY-John Innes Centre-08-07-2020-15:50:15:918-1289
Release date: 2020-09-08, or until publication

### Experimental Model and Subject Details

#### Plant materials, EMS-induced mutagenesis and growth condition

All *Capsella rubella* materials used in the study were in *Cr22.5* ecotype background. The *pHS:CRE/BOB-lox* line was described in [6], the *pDR5v2:GUS*, *pCrYUC9:GUS* and *pCrTAA1:GUS* reporter lines were previously described [8]. All these reporters were introgressed into *htb-1* mutant by crossing.

For mutant screening, wild-type (WT) *Cr22.5* seeds were incubated with ethyl methanesulphonate (EMS, Sigma) at a concentration of 0.25% by volume in 0.02% Tween-20 (Sigma) rotating for 16 hours followed by 12 washes in 0.02% Tween-20 - in water. The seeds were germinated on soil in long-day (16 hr light/8 hr dark) conditions at 22°C and harvested to generate the M2 population. The *htb-1* mutant was discovered in the M2 segregation population. The *htb-1* mutant was backcrossed to WT three times to wash the genetic background and used for further studies.

The seeds were germinated on MS medium 1% sucrose and 0.8% agar containing 10 μM Gibberellin (Sigma) at 22°C. The 10-day-old seedlings were then transplanted into soil in a controlled environment room at 22°C, 16 hr light/8 hr dark conditions.

### Method Details

#### Plasmids construction and plant transformation

For the construction of the *pHTB:GUS* reporter plasmid, ~1.6kb promoter of Carubv10008238 was isolated and inserted upstream of *GUS* gene of pCambia1301 vectors. For the construction of *pHTB:HTB:GFP* plasmid, the genomic sequence from the Carubv10008238 locus (~7.9kb) was isolated and inserted into the pCambia1302 vectors. For *pHTB:AtSPF1/ASP1* and *pHTB:HTB* plasmid, the full length of genomic DNA of *AtSPF1/ASP1* (At1g09730,~6.2kb) or *HTB* (~6.5kb) was inserted downstream of the native *HTB* promoter in pCambia1302 vectors. The *pCrIND:CrIND*^*K124R*^*:GFP* plasmid was domesticated from the *pCrIND:CrIND:GFP* plasmid described in [8]. For construction of *pLhGR≫CrIND:FLAG* plasmids, the *CrIND* and *CrIND*^*K124R*^ coding sequence is fused with 3X FLAG and inserted downstream of GR-inducible promoter to generate the *pGAL6:CrIND:FLAG* and *pGAL6*:*CrIND*^*K124R*^:*FLAG* plasmids. The resultant plasmids were recombined with *p35S*:*GVG*:*GR* plasmid and phosphinothricin selection marker using golden-gate cloning methods to produce the binary vectors. For construction of the CRISPR/Cas9 genome editing plasmids, the DNA sequences encoding gRNAs adjacent to the PAM sequences (NGG) were designed using the CRISPR-P 2.0 software [32] that target two specific sites in the first exons of *Carubv10008238*. The gRNAs (Table S1) were synthesized as oligonucleotides with golden-gate cloning adapters and then were insert downstream of U6 promoters. The resulting gRNAs plasmid were then recombined with *pRPS5a:Cas9z:E9t* and hygromycin selection marker using golden-gate cloning methods to produce the binary vectors. All vectors were verified by sequencing and introduced into *Agrobacterium tumefaciens* strain LBA4404 by electroporation.

Transformation of *Capsella* followed the floral dipping method previously described [8]. The transformants were screened on MS plants with 1% sucrose and 0.8% agar containing 40mg/L hygromycin (Roche) or 25mg/L DL-phosphinothricin (Duchefa). For each construct, at least 10 independent transformants were obtained for further analysis.

#### Genome sequencing and association mapping

Leaf materials were collected from the BC_3_F_2_ segregation population of *htb-1* and WT crossing. Samples were pooled as WT and mutant (Mu) based on the fruit phenotypes, with each pool containing ~90 individuals. Nuclear DNA was extracted and fragmented and the sequencing libraries were prepared according to the manufacturer’s instructions (Illumina). Sequencing reactions were processed on Illumina NextSeq500 platform generating paired-end reads with 100 X in depth. The SNPs were extracted by aligning the sequencing results with the v.1.0 reference genome of *Capsella rubella* [35]. For association mapping, we filter the SNPs between the WT and Mu samples by three criteria: (1) only consider the G to A and C to T SNPs as these are the mutations induced by EMS; (2) the mutation is heterozygous in the WT pool and homozygous in the Mu pool; (3) the mutated SNP frequency in WT pool is 33.3% and 100% in the Mu pool. From such screen, we identified two candidates, *Carubv10012951* and *Carubv10008238*. The G to A mutation happened in the first exon of *Carubv10012951* generates a synonymous mutation that preclude it for further consideration.

#### Phenotyping and Scanning Electron Microscopy (SEM)

The shoulder index value was calculated with the anti-trigonometric function θ = Arctan((L1-L2)/W) using the parameters described previously [8]. For whole-mount fruit photos, stage 17 fruits of each genotype were collected and photographed using Nikon D610 camera with a 105mm prime lens. For Scanning Electron Microscopy (SEM), the inflorescences of each genotype were fixed in FAA and dissected. The samples were critically-point dried in CO_2_ and spotter-coated with gold. The samples were subsequently examined using a Zeiss Supra 55VP field Scanning Electron Microscope with an acceleration voltage of 3.0 kV.

#### Live imaging and cell growth analysis

For live imaging, *pHS:CRE/BOB-lox* lines were grown on soil in a glass house under long-day conditions until bolting (22°C, 16 hr light/8 hr dark). Then, the inflorescences were dipped into water bath at 38°C for 20 min and plants were grown for next 7 days. The fruits at stage 12 were dissected and transferred onto Petri dishes containing 1/2 MS medium including vitamins (Duchefa) supplemented with 1% sucrose. Half of the fruit epidermis was imaged with RFP signal at 48-h intervals using a Leica SP5 upright laser confocal microscope with a water immersion objective (x25/0.95). Excitation wavelengths and emission windows were 514 nm and 529-545 nm. Confocal stacks were acquired at 1024x1024 resolution, with less than 0.5-μm distance in Z-dimension. Between imaging, samples were kept in a growth chamber under long-day condition (22°C, 16 hr light/8 hr dark). The acquired images were stitched and analyzed using MorphoGraphX [16]. In order to calculate the cell area ratio and growth anisotropy, cells showing fluorescence were segmented and cell relations were indicated manually between successive time points. If cells divided in the subsequent time points, the daughter cells were merged. Heat-maps between two time-points are shown on the later time-point (e.g., heatmap for fruit stage 12-13 is shown on the fruit stage 13). Representative growth tracking series were collected from a single growth tracking experiment and 3 time-lapse series were performed for wild-type and *htb-1*.

#### Auxin treatment and auxin metabolite quantification

To quantify auxin metabolite levels in the fruit, shoulder tissues of stage 14 fruit of WT and *htb-1* fruits were dissected under a light microscope and immediately fixed in liquid nitrogen. Extraction, purification and the LC-MS/MS analysis of endogenous IAA and specific IAA metabolites was carried out according to the method described previously [36].

#### RNA extraction, comparative transcriptomic sequencing and expression analysis

Either the whole fruit of stage 13 or the fruit shoulder samples from stage-14 fruits of WT and *htb-1*, respectively, were immediately fixed in liquid nitrogen. Total RNA was isolated from the samples using the RNeasy Plant Mini Kit (QIAGEN). 500ng of total RNA was reverse transcribed into cDNA in a 10 μL reaction with the SuperScript IV First-Strand Synthesis System (ThermoFisher) according to the manufacturer’s instructions.

For RNA-sequencing, poly (A) mRNA was purified from total RNA prepared from stage-13 fruits and fragmentated. Double-strand cDNA was synthesized, followed by sequencing adaptor ligation, electrophoresis purification and PCR amplification to generate the libraries using mRNA-Seq 8 sample prep kit (Illumina) according to the manufacturer’s instructions. The libraries were paired-end sequenced on the Illumina NextSeq sequencer. The clean reads generated by trimming the adapters were mapped and annotated against the *Capsella rubella* v.1.0 genomic sequence using the Kallisto version 0.44.0 [37]. Read counts were generated using Kallisto version 0.44.0 [37]. Differentially expressed genes were identified as those with a fold change ≥ 2 and a *p* value < 0.05 using DESeq2 software in R environment [38] (Data S1). The enrichment of the DEGs in the biological pathways were analyzed with DAVID Bioinformatics Resources 6.8 [39] (Data S1). Two biological replicates of RNA-seq for each sample were conducted.

For real-time qPCR, gene specific primers were designed (Table S1), and verified by PCR and sequencing. The efficiency of the primers (95% to 105%) was determined by creating a standard curve. The SYBR Green JumpStart Taq Ready Mix (Sigma) was used to perform real-time qPCR with ROX as a reference dye on a BioRad CFX96 Q-PCR System (BioRad). The CT value of each gene was determined by normalizing the fluorescence threshold. The relative expression level of the target gene was determined using the ratio = 2^-ΔCT^ method, and *CrUBQ10* was used as an internal control.

For the subcellular localization of the proteins in protoplast. Protoplast preparation and transformation was followed the protocol described in *Arabidopsis* [40] with minor modifications. Briefly, protoplasts were prepared from fully expanded leaves of 3-week old seedlings under short-day growth condition (22°C, 10 hr light/14 hr dark) using enzyme buffer [20mM MES (pH 5.7); 1.5% (wt/vol) cellulase R10 (Yakult); 0.4% (wt/vol) macerozyme R10 (Yakult); 0.4M mannitol; 20mM KCl; 10mM CaCl_2_ and0.1% BSA]. A total of 10 μg plasmid was transformed into 200 μL protoplast containing 2-4 × 10^4^ cells using PEG-mediated transformation. The cells were cultured in W5 buffer [2mM MES (pH 5.7); 154mM NaCl; 125mM CaCl_2_ and 5 mM KCl] in dark condition at 22°C over-night and then subjected to confocal microscope (Leica SP5 laser scanning microscope) examination.

GUS histochemical assay were performed as previously described [8].

#### Chromatin immunoprecipitation (ChIP) and Western Blot (WB)

Stage13-16 fruits from *htb-1/crind-1*^*ge*^ *pCrIND:CrIND*^*K124R*^*:GFP*, *htb-1* and *crind-1*^*ge*^ plants were collected and fixed in 1 x PBS buffer containing 1% formaldehyde under vacuum for 15min. Approximately 3.0 g of tissue was ground in liquid nitrogen and nuclear was isolated by filtering with two layer of miracloth (Merck), chromatin fragments were prepared by sonication. After sonication, a 1/20 sample was taken out as DNA Input. The remaining samples underwent immunoprecipitation. GFP-tagged protein together with the associated DNAs were immunoprecipitated by using Pierce Protein G Magnetic Beads (ThermoFisher) coated with monoclonal anti-GFP antibody (Roche) at 4°C for 2 hr. Beads were washed two times with the immunoprecipitation buffer followed by two washes with TE buffer. Reverse crosslinking was done by boiling the beads at 65°C for 12 hours in presence of 1% SDS followed by Proteinase K treatment at 45°C for 1 hour. DNA was ethanol precipitated following phenol/chloroform extraction. qPCR was performed using SYBR Green JumpStart Taq ReadyMix (Sigma) on a BioRad CFX96 Q-PCR System (BioRad).

For detection of the SUMO-conjugation in the cells, ~0.5-g samples of leaf, inflorescence, stage-13 and stage-15 fruit tissues from WT and *htb-1* were fixed in liquid nitrogen and grinded. Total protein was extracted using extraction buffer [50 mM Tris; 150 mM NaCl; 0.2% (v/v) Triton X-100] supplemented with 1X Complete Protease Inhibitor Cocktail (PI, Roche), 20 mM N-Ethylmaleimide (NEM, Sigma) at 4°C for 1 hour. The supernatants were recovered after two steps of centrifuge at 13000 rpm, 4°C for 15 mins. Equal amounts of protein extracts were loading on a standard SDS-PAGE 10% (w/v) acrylamide gel and separated by electrophoresis. Protein was transferred onto a PVDF membrane (GE Healthcare) using a Mini Trans-Blot Cell (Bio-Rad). The membrane was blocked in blocking solution [5% (w/v) dry milk powder in TBST (1X TBS+0.1% Tween 20)] at 4°C for 4 hr. The primary antibody anti-SUMO1 (Abcam, 1:1000) or anti-α-Tubulin (Sigma, 1:5000) was added and incubated over-night at 4°C. The membrane was washed three times with TBST for 10 mins each step, and then incubated with the secondary anti-rabbit antibody (SUMO1, Abcam, 1:10000) or anti-mouse (α-Tubulin, Abcam, 1:10000) in blocking solution for 2 hr. The membrane was washed as described above and exposed to a film (Kodak) using a chemiluminescence reaction with Pierce ECL Western Blotting Substrate (ThermoFisher).

For detection of the SUMOylation of CrIND, the 7-day old *pLhGR≫CrIND*^*K124R*^*:FLAG* seedlings were treated with 10 μM dexamethasone (DEX) (Sigma) in liquid MS medium supplemented with 1% sucrose at 22°C for 12 hr. ~1.0-g samples of each genotype were fixed and grinded into a fine powder in liquid nitrogen. The proteins were extracted using GTEN buffer [10% Glycerol; 25 mM Tris; 1 mM EDTA; 150 mM NaCl; 0.1% (v/v) NP-40] supplemented with 1X PI, 20 mM NEM, 1 mM PMSF (Roche) and 10 mM DTT at 4°C for 1 hr. The supernatants were collected after two steps of centrifuge at 13000 rpm, 4°C for 15 mins and 50 μL sample was taken out as Input. The remaining lysates were subjected to immune-precipitation using anti-FLAG M2 magnetic beads (Sigma) at 4°C for 2 hr. The beads were then washed with IP buffer [GTEN buffer; 1X PI; 20 mM NEM; 1 mM PMSF and 100 μM DTT] four times at 4°C for 5 mins each step. 10 μL Input and 5 μL IP samples were loaded into a standard 10% acrylamide SDS-PAGE gel. The western blot was conducted according to the aforementioned protocol using either anti-SUMO1 or anti-FLAG (Abcam, 1:5000) antibody.

#### Population genetics, selection test and phylogeny

To test for evidence of selection at the *HTB* gene, we used polymorphism data for 20 individuals from one *Capsella grandiflora* population and double-checked the results with 13 samples collected in different populations [41]. We conducted a McDonald-Kreitman test (dN/dS) to compare the ratio of synonymous (4-fold degenerate) and non-synonymous (0-fold degenerate) polymorphisms (Pn/Ps) in the coding sequence of *CgHTB* within *Capsella* to fixed differences (Ka/Ks) between *Capsella* and *Arabidopsis* [31]. To assess whether the observed values of Ka/Ks and Pn/Ps were unusual, we compared the observed ratios to Ka/Ks and Pn/Ps at the *CgHTB* gene to ratios of genes in genomic regions with comparable recombination rates, gene densities (in 50-kb windows) and similar expression levels. *P*-values of a two-sided test for a difference between observed Ka/Ks and Pn/Ps and expected Ka/Ks and Pn/Ps were calculated based on the distribution of observed Ka/Ks and Pn/Ps of the comparable genes. In addition, we used the direction of selection (DoS) statistic which describes the direction and extent of selection with positive values indicating positive selection and negative values purifying selection, respectively [42].

For the phylogenetic analysis of ULP family of Cysteine Proteases from *Arabidopsis* and *Capsella*, the full-length protein sequences were downloaded from phytozome database and aligned with Clustal X software [33]. The Neighbor-Jointing (NJ) tree with bootstrap support value was generated based on Protein sequence using MEGA5 software [34].

### Quantification and Statistical Analysis

All statistics were calculated in Microsoft Excel. All measured data are presented as means ± SD specified along with sample sizes (n) in the methods and in figure legends. Comparisons between groups for the analysis of qRT-PCR and fruit characters was performed with Microsoft Excel Student’s t test, and significance levels are marked as: ^∗^p < 0.05, ^∗∗^p < 0.01.

## Supplemental Information

Document S1. Figures S1–S4 and Table S1 ▪▪▪

Data S1. Differentially Expressed Genes (DEGs) between WT and *htb-1* of Stage-13 Fruits, Related to Figure S3 and STAR Methods (A) List of upregulated DEGs between WT and *htb-1* of stage-13 fruits. (B) List of downregulated DEGs between WT and *htb-1* of stage-13 fruits. Data were obtained from two biological replicates.

Data S2. Gene Ontology (GO) and Pathway-Enrichment Analyses of DEGs Identified between WT and *htb-1* of Stage 13 Fruits, Related to Figure S3 and STAR Methods (A) Pathways enriched among upregulated DEGs in stage-13 fruits of *htb-1* compared with WT. (B) Pathways enriched among downregulated DEGs in stage-13 fruits of *htb-1* compared with WT. Data were obtained from two biological replicates.

Document S2. Article plus Supplemental Information
