# Supplementary material for: HEARTBREAK Controls Post-translational Modification of INDEHISCENT to Regulate Fruit Morphology in Capsella
Source: Curr Biol. 2020 Oct 5;30(19):3880–3888.e5. doi: 10.1016/j.cub.2020.07.055 (PMC7544509; doi:10.1016/j.cub.2020.07.055)
Supplement: Document S1. Figures S1–S4 and Table S1 — ▪▪▪ [file mmc1.pdf]

Current Biology, Volume *30*

Supplemental Information

HEARTBREAK Controls Post-translational Modification of INDEHISCENT to Regulate Fruit Morphology in *Capsella*

Yang Dong, Mateusz Majda, Jan Šimura, Robert Horvath, Anjil K. Srivastava, Łukasz Łangowski, Tilly Eldridge, Nicola Stacey, Tanja Slotte, Ari Sadanandom, Karin Ljung, Richard S. Smith, and Lars Østergaard

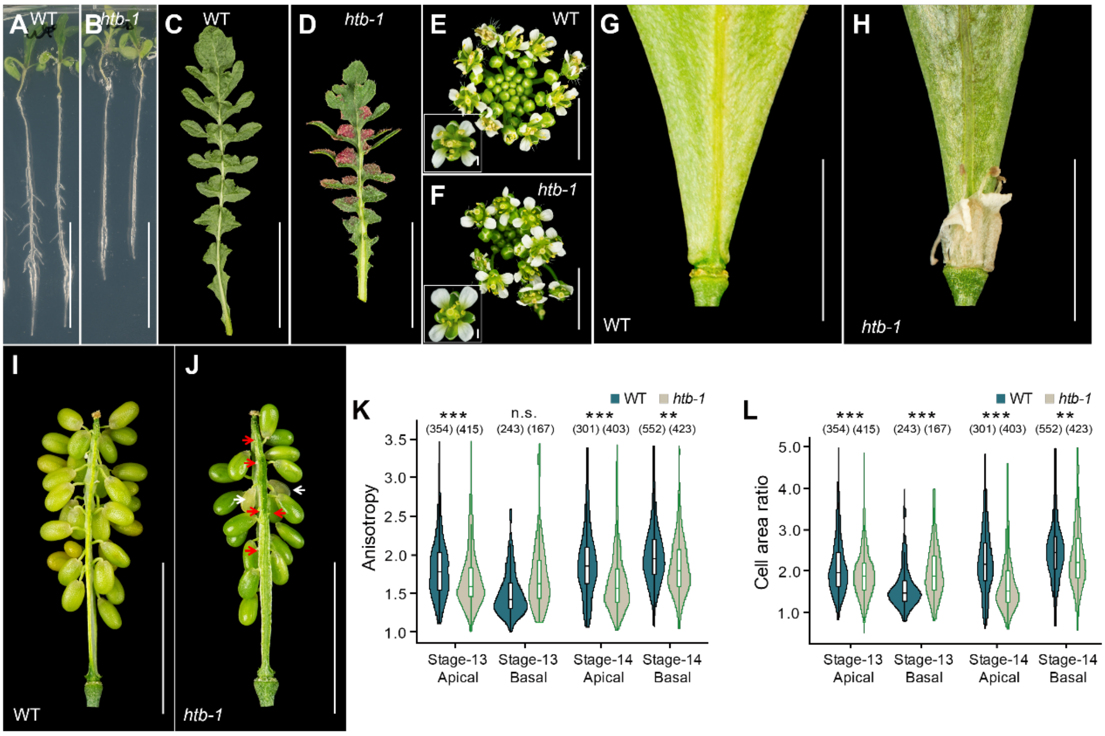

Figure S1. *htb* mutant exhibits pleiotropic defects in development and compromised cell growth in the fruit valves, Related to Figure 1.

(A and B) Roots of 7 days old seedlings showing lateral root growth of *htb-1* (B) was suppressed compared with WT (A). (C and D) The sixth leaves of 21 days-old seedlings showing reduced growth and over accumulation of anthocyanin in *htb-1* (D) compared with WT (C). (E and F) Top view of inflorescence showing *htb-1* (F) exhibits more compact inflorescence and bigger flower organs compared with WT (E). (G and H) Basal part of a mature fruits (stage 17b) showing abscission defect of flower organs in *htb-1* (H) compared with WT (G). (I and J) Opened siliques of stage-17 fruits showing ovules that remain unfertilized (red arrows) and ovules that are fertilized but arrested in development (white arrows) in *htb-1* (J), but not observed in WT (I). (K) Quantification of cell anisotropy between wild-type (WT) and *htb-1* in the apical and basal part of the fruit. (L) Quantification of cell area ratio between wild-type (WT) and *htb-1* in the apical and basal part of the fruit. The number in parentheses indicate the cells for quantification. Error bars represent SD. ** $p < 0.01$, *** $p < 0.001$ (Student's t test). Scale bars, (A)-(D), 2 cm; (E) and (F), 0.5 cm; insertion in (E) and (F), 500 µm; (G) and (H), 2 mm; (I) and (J), 5 mm.

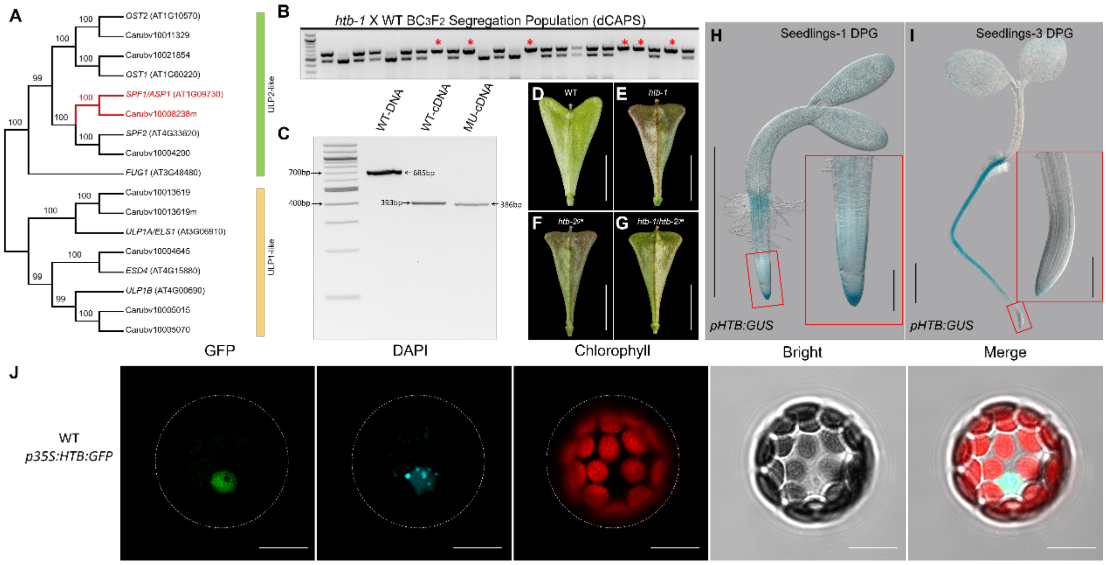

Figure S2. Expression analysis of *HTB* during development, Related to Figure 2. (A) Neighbor-joining tree of proteins encoded by the ULP family of Cysteine Proteases genes from the Capsella and Arabidopsis genome, bootstrap values over 50% (1,000 replicates) are indicated for each branch, the orthology of Carubv10008238 and *SPF1/ASP1* is indicated by red branches. (B) A genotyping analysis of the BC3F2 segregation population showing the *htb* mutation segregates as a single-locus recessive trait, red asterisks indicate the *htb* homozygous lines. (C) RT-PCR of the WT and *htb-1* allele showing the 7-bp deletion is readily detected. (D-G) Fruit morphology of WT (D), *htb-1* (E), *htb-2^{ge}* (F), F1 of *htb-1* and *htb-2^{ge}* cross (G) at stage 17. (H) In seedlings 1 Day Post Germination (1DPG), *HTB* expression is detected in the roots, root tips and tip of the cotyledons. (I) In 3DPG seedlings, *HTB* expression is detected in the roots, root tips and vascular tissues in the cotyledons. (J) Subcellular localization of HTB:GFP protein in WT protoplast cells transiently expressing the *p35S:HTB:GFP* plasmid.. Scale bars, (D)-(G), 5mm; (H) and (I), 1 mm; insertions in (H) and (I), 100 μm; (J), 60 μm.

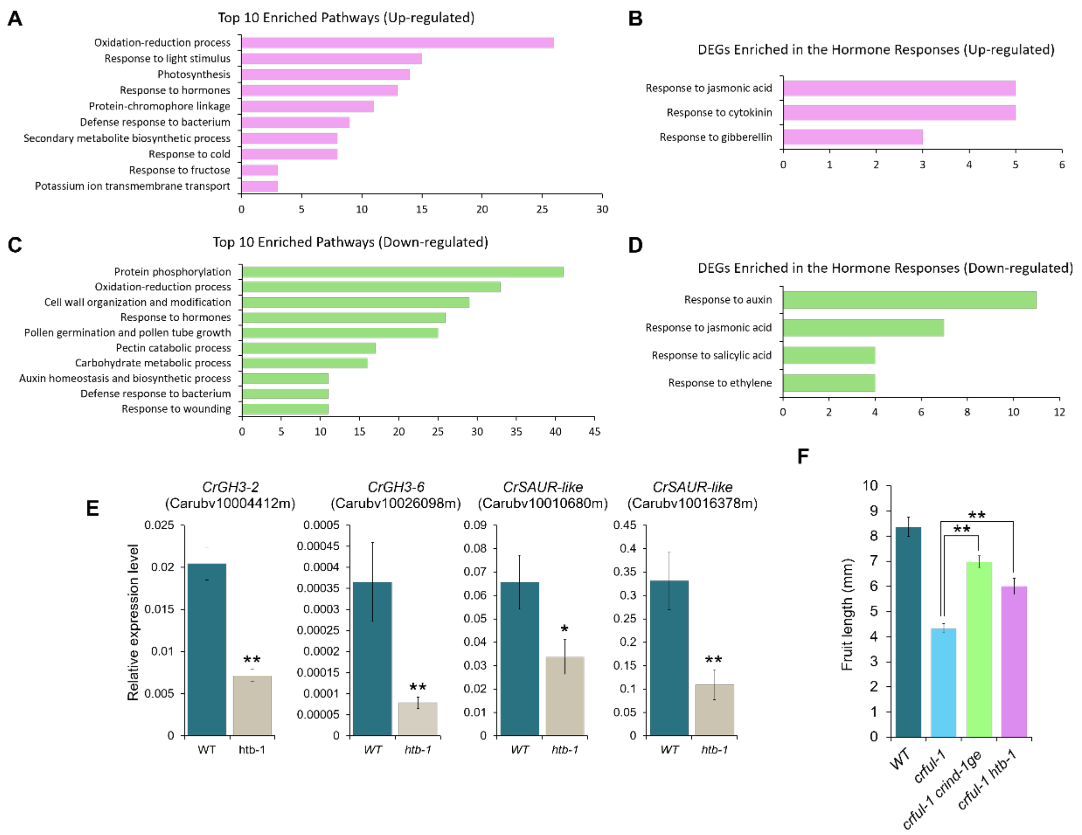

Figure S3. HTB controls fruit shape development via regulating auxin biosynthesis, Related to Figure 3.

(A) Top 10 pathways enriched among up-regulated differentially expressed genes (DEGs) in stage-13 fruits of *htb-1* compared with WT. (B) Categories of up-regulated DEGs involved in hormone signalling. (C) Top 10 pathways enriched among down-regulated DEGs in stage-13 fruits of *htb-1* compared with WT. (D) Categories of down-regulated DEGs involved in hormone signalling. (E) qRT-PCR analysis of four representative genes identified as down-regulated DEGs involved in auxin signalling using independent RNA samples. (F) Fruit size of different mutant combinations. Fruit length was quantified as values from the stigma to the pedicel. Error bars in (E) represent SD of three biological replicates; (F) represent SD of 30 individual fruits. **p*< 0.05, ***p* < 0.01 (Student's t test).

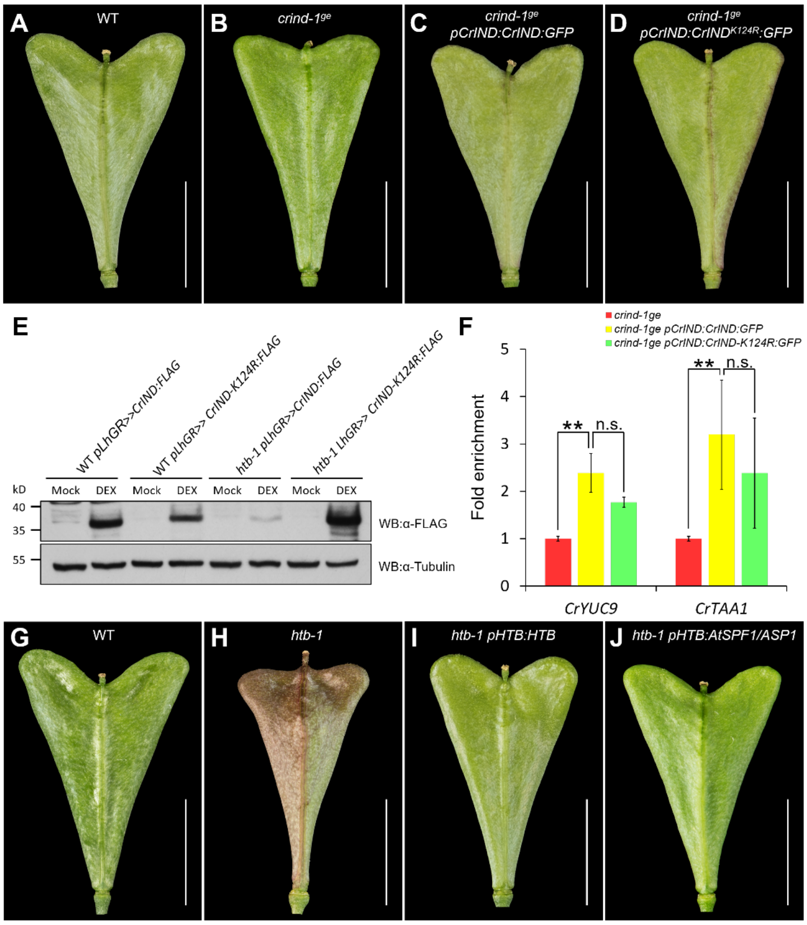

K

Samples	Ka/Ks	Pn/Ps	*p*-value	DoS	dN/dS
CgHTB-Population (n=20, pn=1)	1.660	1.833	0.864	-0.023	0.292
CgHTB-Mixed Population (n=13, pn=13)	1.660	2.308	0.462	-0.074	0.292

Figure S4. Molecular and morphological effects of CrIND and CrIND^{K124R} on fruit shape determination and evolutionary conservation of HTB-homologs on fruit development, Related to Figure 4.

(A-D) Fruit morphology at stage 17 of WT (A), *crind-1^{ge}* (B), *crind-1^{ge} pCrIND:CrIND:GFP* (C), and *crind-1^{ge} pCrIND:CrIND^{K124R}:GFP* (D). (E) Immunoblot of the proteins extracted from *pLhGR>>CrIND:FLAG* and *pLhGR>>CrIND^{K124R}:FLAG* plants with anti-FLAG showing CrIND:FLAG/CrIND^{K124R}:FLAG protein accumulates in response to 12 hrs of DEX treatment, the same plants were mock treated with ethanol showing no expression of CrIND:FLAG/CrIND^{K124R}:FLAG proteins. The α-tubulin was immunoblotted as a loading control. (F) Chromatin Immuno-Precipitation (ChIP) analysis of CrIND/CrIND^{K124R} associated with the *CrYUC9* and *CrTAA1* promoters. (G-J) Fruit morphology at stage 17 of WT (G), *htb-1* (H), *htb-1 pHTB:HTB* (I), and *htb-1 pHTB:AtSPF1/ASP1* (J). (K) Selection test of *HTB* orthologs in *Capsella grandiflora* population. n, number of individuals and pn, number of populations. Divergence statistics refer to divergence between *Capsella* and *Arabidopsis*. *p*-values of a two-sided test for a difference between observed Ka/Ks and Pn/Ps and expected Ka/Ks and Pn/Ps were calculated based on the distribution of observed Ka/Ks and Pn/Ps of the comparable genes. Scale bars in (A)-(D) and (G)-(J) represent 5 mm. Error bars in (F) represent SD of three biological replicates. **$p < 0.01$ (Student's t test).

**Primer**	**Sequence (5’ to 3’)**	**Experiment**
HTB-gRNA1	GTTCAGAAACAAGAACCTGA	CRISPR/Cas9 Gene Editing
HTB-gRNA2	AACTAGTCTCACGTTTCGTT	CRISPR/Cas9 Gene Editing
HTB-geno-F	CTTGGATTCTCCGTGTACCG	Genotyping
HTB-geno-R	AACGTGAGACTAGTTCTGGACTCC	Genotyping
pHTB-GUS-F	CCTCTAGAGTCGACCTGCAGAGAAAAAACAGGATTCAATAC	Plasmid Construction
pHTB-GUS-R	CTCAGATCTACCATGGCAACACAGAAATCTGAGGACA	Plasmid Construction
pHTB:HTB:GFP-F	ATCCTCTAGAGTCGACAGAAAAAACAGGATTCAATACTGG	Plasmid Construction
pHTB:HTB:GFP-R	GTCAGATCTACCATGGAAGTCTTCTCGATCTCATC	Plasmid Construction
gAtSPF1/ASP1-F	CCTCAGATTTCTGTGTTGATGAAGAAAAACTTTGAAGTAT	Plasmid Construction
gAtSPF1/ASP1-R	GTCAGATCTACCATGGCTATTTCTCCATCTCCTCAG	Plasmid Construction
pHTB:HTB-F	ATCCTCTAGAGTCGACAGAAAAAACAGGATTCAATACTGG	Plasmid Construction
pHTB:HTB-R	GTCAGATCTACCATGGCTTAGTCTTCTCGATCTCATC	Plasmid Construction
p35S:HTB:GFP-F	GGACTCTTGACCATGGGCTAGCATTTGTTGTTGACTTG	Plasmid Construction
p35S:HTB:GFP-R	GTCAGATCTACCATGGAAGTCTTCTCGATCTCATCA	Plasmid Construction
pLhGR>>CrIND$^{(K124R)}$*:FLAG-F*	TGAAGACTTAATGATGGAGCCTCAACCTCA	Plasmid Construction
pLhGR>>CrIND$^{(K124R)}$*:FLAG-R*	TCACTTATCGTCATCGTCCTTATAATCCGCTGCTGCAGCGTCGAGGTCATGGTCCTTATAGTCTCCGTCATGGTCCTTATAATCGGTTTGGGAGTTGTGGTAATAACAA	Plasmid Construction
pCrIND:CrIND$^{(K124R)}$*:GFP-F*	ATCCTCTAGAGTCGACGATGCCTAAATTAGCTTTTGATG	Plasmid Construction
pCrIND:CrIND$^{(K124R)}$*:GFP-R*	GTCAGATCTACCATGGAGGTTTGGGAGTTGTGGTAAT	Plasmid Construction

Primer	Sequence (5' to 3')	Experiment
CrGH3.2 (Carubv10004412m)-rt-F	GAAAGATATAAAGCCGATTG	Gene Expression
CrGH3.2 (Carubv10004412m)-rt-R	AAATTTATTATACGATTTAAGAACC	Gene Expression
CrGH3.6 (Carubv10026098m)-rt-F	GAAACTTCTGAGGAAGAAGG	Gene Expression
CrGH3.6 (Carubv10026098m)-rt-R	AAGCGAAATTAAAATTAAGA	Gene Expression
CrSAUR (Carubv10010680m)-rt-F	TATTTTTTCTTGGTGGATGA	Gene Expression
CrSAUR (Carubv10010680m)-rt-R	GTTTAATAGATCACTCTTAGAGGCA	Gene Expression
CrSAUR (Carubv10016378m)-rt-F	ATTTAGGACTCTGATGGATG	Gene Expression
CrSAUR (Carubv10016378m)-rt-R	CTTCTTGTTACACGACATGTAC	Gene Expression

Table S1. Primers used in this study. Related to STAR Methods.
